# Structural basis of properties, mechanisms, and channelopathy of cyclic nucleotide-gated channels

**DOI:** 10.1080/19336950.2023.2273165

**Published:** 2023-10-31

**Authors:** Zhengshan Hu, Jian Yang

**Affiliations:** Department of Biological Sciences, Columbia University, New York, NY, USA

**Keywords:** CNG channel, cryo-EM structure, allosteric gating, cGMP, conformation landscape

## Abstract

Recent years have seen an outpouring of atomic or near atomic resolution structures of cyclic nucleotide-gated (CNG) channels, captured in closed, transition, pre-open, partially open, and fully open states. These structures provide unprecedented molecular insights into the activation, assembly, architecture, regulation, and channelopathy of CNG channels, as well as mechanistic explanations for CNG channel biophysical and pharmacological properties. This article summarizes recent advances in CNG channel structural biology, describes key structural features and elements, and illuminates a detailed conformational landscape of activation by cyclic nucleotides. The review also correlates structures with findings and properties delineated in functional studies, including nonselective monovalent cation selectivity, Ca^2+^ permeation and block, block by L-*cis*-diltiazem, location of the activation gate, lack of voltage-dependent gating, and modulation by lipids and calmodulin. A perspective on future research is also offered.

## Introduction

Cyclic nucleotide-gated (CNG) channels belong to the voltage-gated ion channel (VGIC) superfamily, which includes, among others, voltage-gated K^+^, Na^+^, and Ca^2+^ channels and transient receptor potential channels [1]. They are also members of the cyclic nucleotide-binding domain (CNBD) channels, which in addition includes hyperpolarization-activated, cyclic-nucleotide gated (HCN) channels and the ether-à-go-go-type (KCNH) channels [2]. CNG channels are essential for vision and smell in vertebrates and also play an important role in chemosensation in invertebrates [3–6]. In vertebrate rod and cone photoreceptors, light activation of photopigments decreases intracellular cyclic guanosine monophosphate (cGMP) concentration and closes cGMP-sensitive CNG channels, resulting in membrane hyperpolarization. In olfactory sensory neurons, odorant activation of olfactory receptors increases intracellular cyclic adenosine monophosphate (cAMP) concentration and opens cAMP-sensitive CNG channels, leading to membrane depolarization. CNG channels are also expressed in the central nervous system (CNS), where they regulate neuronal excitability, synaptic transmission and plasticity, development, neurogenesis, and pain processing [4,6]. Numerous inherited mutations in both rod and cone photoreceptor CNG channel genes have been linked to degenerative visual disorders such as retinitis pigmentosa and achromatopsia [4,6–8].

There are six vertebrate CNG channel subunits: CNGA1–CNGA4, CNGB1, and CNGB3 [4,6]. CNGA1, CNGA2, and CNGA3 are principal subunits that can form functional homomeric channels. CNGA4, CNGB1, and CNGB3, however, do not form functional homomeric channels on their own, but they assemble with the proper principal subunits to form functional heteromeric channels. Rod CNG channels are formed by CNGA1 and CNGB1, cone CNG channels by CNGA3 and CNGB3, and olfactory CNG channels by CNGA2, CNGA4, and CNGB1b. CNGB subunits profoundly affect the biophysical properties and regulation of their respective heteromeric channels. CNG channel subunits have also been identified in fruit flies, nematodes, plants, and bacteria.

CNG channels are tetramers comprised four identical or different subunits. Each subunit contains six transmembrane segments (S1-S6), a pore-loop and a pore helix between S5 and S6, and cytoplasmic amino (N) and carboxyl (C) termini. The pore-loop forms the ion selectivity filter (SF), and S6 forms the rest of the pore. S5, the pore loop and pore helix, and S6 constitute the pore domain (PD). S4 contains regularly spaced positively charged amino acids, a common feature found in S4 of typical voltage-gated K^+^, Na^+^, and Ca^2+^ channels. S1-S4 segments interact and constitute the voltage-sensor domain (VSD) or voltage sensor-like domain (VSLD). A C-terminal domain called the C-linker is connected to S6 via a short linker. Immediately following the C-linker is the CNBD.

Vertebrate CNG channels are nonselective cation channels, permeable to Na^+^, K^+^, and Ca^2+^. Ca^2+^ also blocks monovalent cation permeation. Although having an S4 resembling that of typical voltage-gated K^+^ channels in amino acid sequence, vertebrate CNG channels are not activated by voltage; they are activated only by intracellular cGMP or cAMP. CNG channels are inhibited by L-*cis*-diltiazem, Ca^2+^/calmodulin, and certain lipids.

This review focuses on structural studies of CNG channels and how these studies provide mechanistic insights into CNG channel properties and mechanisms, complementing recent reviews on structural mechanisms of CNBD channels [2] and photoreceptor CNG channels [9]. Readers interested in CNG channel molecular biology, cell biology, physiology, pharmacology, biophysics, regulation, and channelopathy are referred to the many reviews that cover these topics, only some of which are cited here [3–8,10–12]. HCN and KCHN channels are not reviewed in this article. Although these channels and CNG channels share some common molecular features, they differ markedly in some key properties. For example, HCN channels have a distinct feature of being activated by membrane hyperpolarization, and although cAMP binds HCN channels and modulates their activities, it does not activate the channels on its own. KCNH channels are K^+^-selective channels, and they do not bind and are not regulated by cyclic nucleotides. Readers interested in advances in structural mechanisms of HCN and KCNH channels are referred to recent reviews on these topics [2,13–15].

## Timeline of structural studies

Structural biology of CNG channels actually started with the determination of X-ray crystal structures of the CNBD and C-linker of HCN2 bound with cAMP or cGMP, as the CNBD and C-linker regions of HCN and CNG channel subunits are highly similar in primary sequence [16]. These structures show that the CNBD of HCN channels has the same fold as the CNBD of other cyclic nucleotide-binding proteins and reveals for the first time that the C-linker tetramerizes. After a long pause, crystal structures were obtained of C-terminal fragments of CNGA1 and CNGA3 subunits containing a coiled-coil domain that is critical for the proper assembly of CNGA/CNGB channels [17] (Table 1). Efforts were undertaken in parallel to determine the structures of isolated domains or full-length proteins of MloK1 (also named MlotiK1), a bacterial cyclic nucleotide-gated K^+^ channel, by X-ray crystallography and cryogenic electron microscopy (cryo-EM) [18–23] (Table 1). Since MloK1 differs greatly from vertebrate CNG channels in several aspects (e.g. it is K^+^ selective, its C-linker is much shorter, and it has a canonical domain-swapped VSLD), it is not further discussed in this review.

**Table 1.** Select information on CNG channel structures.

Year	Channel	Region	Species	Method	Resolution	Reconstitution condition	Condition & State	PDB accession no.	Citation no.
2004	MloK1 WT & R348A	CNBD	Mesorhizobium loti	X-ray crystallography	1.70–2.70 Å	Soluble	With cAMP & apo	1VP6, 1U12	[18]
2007	MloK1	Full-length	Mesorhizobium loti	Electron crystallography	16 Å	Detergent (DM)	With cAMP		[19]
2008	MloK1 WT, R307E & R307W	CNBD	Mesorhizobium loti	X-ray crystallography	2.00–2.90 Å	Soluble	With cGMP/cAMP & apo	3CL1, 3CLP, 3CO2	[20]
2008	MloK1	TM domain	Mesorhizobium loti	X-ray crystallography	3.10 Å	Detergent (LDAO)	Closed	3BEH	[21]
2014	MloK1 T284S/V288S/A352D	CNBD	Mesorhizobium loti	X-ray crystallography	1.25 Å	Soluble	With cGMP	4MUV	[22]
2014	MloK1	Full-length	Mesorhizobium loti	X-ray crystallography	7.00 Å	Detergent (DM)	With cAMP & apo	4CHV, 4CHW	[23]
2011	CNGA1 & CNGA3	CLZ domain	Bos taurus, Homo sapiens	X-ray crystallography	1.90–2.14 Å	Soluble		3SWF, 3SWY	[17]
2017	TAX-4	Full-length	Caenorhabditis elegans	Single particle cryo-EM	3.50 Å	Amphipol (A8–35)	With cGMP, open	5H3O	[24]
2017	LliK	Full-length	Leptospira licerasiae	Single particle cryo-EM	4.20 Å	Detergent (LMNG)	With cAMP, closed	5V4S	[25]
2018	LliK	Full-length	Leptospira licerasiae	Electron crystallography	4.50 Å	Lipid/detergent mixture (DM + *E.coli* polar extract)	With cAMP, open	6EO1	[26]
2018	SthK	Full-length	Spirochaeta thermophila	Single particle cryo-EM	3.35–3.46 Å	Lipid nanodisc (MSP1E3 + POPG)	Apo, with cGMP/cAMP, closed	6CJQ, 6CJT, 6CJU	[27]
2020	TAX-4 WT & F403V/V407A	Full-length	Caenorhabditis elegans	Single particle cryo-EM	2.50–2.70 Å	Lipid nanodisc (MSP2N2 + soybean lipids)	Without cGMP, closed & with cGMP, open	6WEJ, 6WEK, 6WEL	[28]
2021	CNGA1 WT & E365Q	N-terminal truncated	Homo sapiens	Single particle cryo-EM	2.60–3.60 Å	Detergent (digitonin)	Apo closed & cGMP-bound open	7LFT, 7LFW, 7LFY, 7LFX, 7LG1	[29]
2021	CNGA1/CNGB1	Full-length	Bos taurus	Single particle cryo-EM	3.40 Å	Lipid/detergent mixture (CHAPS + soybean lipids)	Apo closed	7O4H	[30]
2022	SthK Y26F & R120A	Full-length	Spirochaeta thermophila	Single particle cryo-EM	2.90–4.30 Å	Lipid nanodisc (MSP1E3 + DOPC/POPG/Cardi olipin)	With cAMP, closed, intermediate, & open	7RSH, 7RTF, 7RTJ, 7RU0, 7RYR, 7RYS	[31]
2022	SthK	Full-length	Spirochaeta thermophila	Single particle cryo-EM	2.41–3.60 Å	Detergent (DDM), lipid nanodisc (MSP1E3 + DOPC/POPA)	Apo closed, cAMP- bound closed, and cAMP-bound open	7TJ5, 7TJ6, 7TKT	[32]
2022	CNGA1/CNGB1	N-terminal truncated	Homo sapiens	Single particle cryo-EM	2.61–3.31 Å	Detergent (digitonin)	Apo closed, cAMP- bound closed, cGMP-bound partial open, L-*cis*- diltiazem bound closed and open	7RH9, 7RHG, 7RHH, 7RHI, 7RHJ, 7RHK, 7RHL	[33]
2022	CNGA3/CNGB3	Full-length	Homo sapiens	Single particle cryo-EM	2.93 Å	Detergent (GDN)	Apo closed	7RHS	[34]
2022	TAX-4 R421W	Full-length	Caenorhabditis elegans	Single particle cryo-EM	2.90–3.20 Å	Lipid nanodisc (MSP2N2 + soybean lipids)	Apo closed, apo open, with cGMP open	7N15, 7N16, 7N17	[35]
2023	CNGA1/CNGB1	Full-length	Bos taurus	Single particle cryo-EM	2.76 Å	Lipid/detergent mixture (CHAPS + soybean lipids)	CaM bound closed	8BX7	[36]
2023	CNGA3/CNGB3	Full-length or *N*- terminal truncated	Homo sapiens	Single particle cryo-EM	3.11–3.60 Å	Detergent (GDN), lipid nanodisc (MSP2N2 + POPC/POPG)	With cGMP, closed, intermediate, & open	8ETP, 8EU3, 8EUC, 8EV8, 8EV9, 8EVA, 8EVB, 8EVC	[37]

The pace of CNG channel structural biology quickened after the “resolution revolution” of cryo-EM [38,39], which enabled the determination of an increasing number of cryo-EM structures of full-length or truncated eukaryotic and prokaryotic CNG channels [24–37] (Table 1). In 2017, a cGMP-bound open-state structure of TAX-4, a CNG channel in the nematode *C. elegans*, was reported [24]. This was the first high-resolution structure of a full-length eukaryotic CNG channel. Soon after, an open-state structure and a closed-state structure were obtained, respectively, for Llik and SthK, two K^+^-selective bacterial CNG channels [25,27]. In 2020 high-resolution structures of TAX-4 were obtained in both apo closed and cGMP-bound open states [28]. The next year high-resolution structures of human rod CNGA1 were also obtained in both apo-closed and cGMP-bound open states [29]. In 2022 structures of a mutant TAX-4 carrying a disease-associated mutation (DAM) were obtained in the absence and presence of cGMP, showing that the mutant channel is spontaneously open [35]. Up till then, all the solved structures were those of homomeric CNG channels. In 2022 three groups simultaneously solved the apo closed-state structures of native heteromeric CNG channels, including bovine rod CNGA1/CNGB1 [30], human rod CNGA1/CNGB1 [33], and human cone CNGA3/CNGB3 [34]. Partially open structures of human rod CNGA1/CNGB1 were also obtained [33]. Meanwhile, strategically placed mutations or reconstitution in appropriate lipids enabled the determination of pre-open, partially open, and fully open structures of SthK [31,32]. In 2023 structures of multiple intermediate states as well as a closed state and a fully open state of human cone CNGA3/CNGB3 were captured, all with fully bound cGMP [37], and a structure of bovine rod CNGA1/CNGB1 in complex with Ca^2+^/calmodulin was obtained [36].

## Structural features and elements

### Subunit stoichiometry

**Figure 1.**
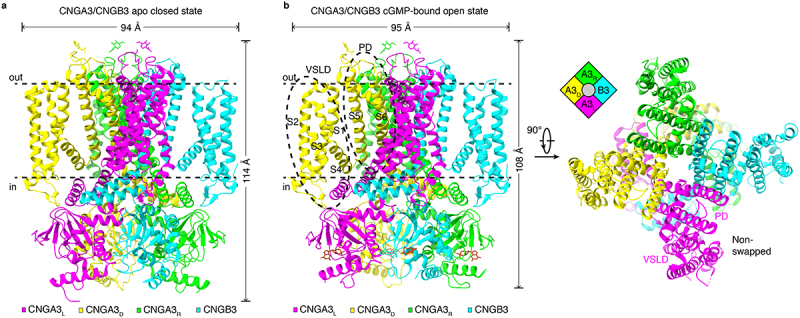
Cryo-EM structures of human CNGA3/CNGB3 in apo closed state and cGMP-bound open state. **a**, apo closed-state structure, viewed parallel to the membrane (PDB accession no. 7RHS). The four subunits are color coded. In reference to the position relative to CNGB3, the CNGA3 subunits are named CNGA3_L_ or A3_L_ (left), CNGA3_D_ or A3_D_ (diagonal), and CNGA3_R_ or A3_R_ (right), respectively. The width and height of the structure are indicated. **b**, cGMP-bound open-state structure, viewed parallel to the membrane or perpendicular to the membrane from the extracellular side (PDB accession no. 8EVC). Inset is a schematic of the subunit arrangement. The voltage sensor-like domain (VSLD) and pore domain (PD) are indicated.

All prokaryotic and eukaryotic CNG channels whose structures have been obtained to date (other than MloK1) show an identical structural architecture: they are tetramers consisting of four identical or different subunits, and their VSLDs and PDs have a non-domain-swapped configuration. Since vertebrate native CNG channels are heterotetramers rather than homotetramers, we will use the structures of the human cone CNGA3/CNGB3 channel as examples throughout this review to describe structural features, elements, and mechanisms. Figure 1 shows the structures of CNGA3/CNGB3 in an apo closed-state and a cGMP-bound open-state [34,37]. The channel is composed of three CNGA3 subunits and one CNGB3 subunit. In these and subsequent structures, the CNGA3 subunits are named CNGA3_L_ or A3_L_ (L for left), CNGA3_D_ or A3_D_ (D for diagonal), and CNGA3_R_ or A3_R_ (R for right), respectively, in reference to their position relative to CNGB3 and following a nomenclature used for CNGA1/CNGB1 structures [33]. Structures of bovine and human rod CNGA1/CNGB1 channels show that they are made up of three CNGA1 subunits and one CNGB1 subunit [30,33]. For the rod CNG channel, the structures confirm the 3:1 subunit stoichiometry concluded from previous biochemical, functional, fluorescence, and structural studies [17,40–42]. For the cone CNG channel, there was an uncertainty about its subunit stoichiometry, with competing results and proposals of a 2 CNGA3:2 CNGB3 stoichiometry [6,43,44] and a 3 CNGA3:1 CNGB3 stoichiometry [7,17,45]. The structures of CNGA3/CNGB3 unambiguously show a 3 CNGA3:1 CNGB3 stoichiometry [34,37].

If CNGA1 and CNGA3 subunits can form functional homotetrameric channels, why do they assemble with CNGB1 and CNGB3 subunits, respectively, in a 3:1 stoichiometry and do so preferentially when the two subunits are co-expressed? The answer lies in the existence of a C-terminal leucine zipper (CLZ) domain in CNGA1 and CNGA3 subunits. This domain follows the CNBD with a short linker and is absent in CNGB1 and CNGB3 subunits. The CLZ domain forms a parallel three-helix coiled-coil trimer [17,40]. Disruption of this trimer alters the preferential 3:1 stoichiometric assembly of CNGA/CNGB channels [17,46]. The CLZ domain coiled-coil trimer is also observed in one of the structures of CNGA1/CNGB1 and is shown to interact with a C-terminal α helix of CNGB1 [33]. Two models have been proposed for the preferential 3:1 assembly of CNGA/CNGB channels: (1) CNGA subunits preferentially form trimers, resulting in a marked increase in the ratio of CNGB/CNGA monomers and by mass action causing CNGB monomers to associate with CNGA trimers [46]; (2) CNGB monomers have a high affinity for CNGA trimers and hence are preferentially incorporated to form CNGA/CNGB heterotetramers [17]. There are biochemical and functional results that are consistent with the second model [17], but the two models are not mutually exclusive.

Homomeric CNG channel structures have four-fold symmetry, but heteromeric CNGA/CNGB channels have no symmetry because of the 3:1 stoichiometry. Thus, the ion conduction pathway of CNGA/CNGB channels is asymmetric in many key regions, including the SF and activation gate. Moreover, because each subunit of CNGA/CNGB channels has different and unique interactions with its adjacent subunits, the four subunits undergo different conformational changes during ligand gating. These points are described in more detail later.

### Non-domain-swapped architecture

A structural feature common to many (but not all) members of the VGIC superfamily is that the VSD (or VSLD) of one subunit (or pseudosubunit) associates with the PD of an adjacent subunit (or pseudosubunit) in a domain-swapped architecture, typically with a long α helical S4-S5 linker connecting the VSD and PD. However, the VSLD of CNG channels interacts only with the PD of the same subunit in a non-domain-swapped configuration (Figure 1b). In this configuration, the VSLD and PD of the same subunit are connected by a short (three amino acids) S4-S5 loop and are positioned close together. The functional importance of a non-domain-swapped VSLD/PD configuration remains to be elucidated, but it is likely related to CNG channel assembly and ligand gating. Non-domain-swapped topology has also been observed in other CNBD and non-CNBD VGICs [2,13,15,47].

### Protomer structure

**Figure 2.**
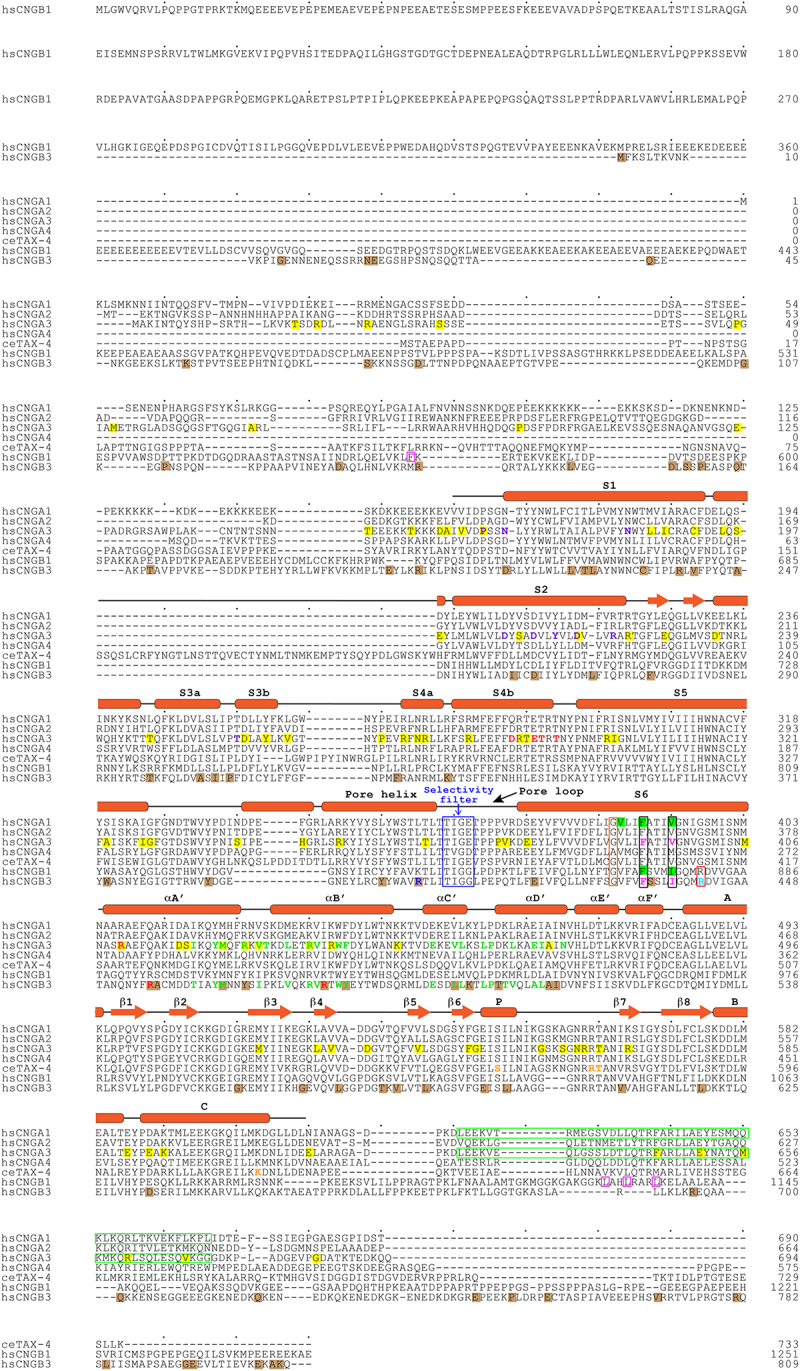
Amino acid sequence alignment of human CNG channel subunits and *C. elegans* TAX-4 subunit, annotated with secondary structures and key functional regions and amino acids. Secondary structures are indicated based on CNGA3/CNGB3 structures. The SF is boxed in blue. The cavity gate and arginine gate are boxed in black and red, respectively, with the cavity gate-forming residues in CNGA3 and CNGB3 shown in magenta, and the arginine gate-forming R442 in CNGB3 shown in cyan. The S6 hinge residue (G388 in CNGA3) is boxed in orange. SF-projecting R403 in CNGB3 is shown in blue. S1-S3 residues involved in interactions with S4 positive charges in CNGA3 are shown in purple. Residues involved in gating ring/TMD interactions in CNGA3 and CNGB3 are shown in red. Residues involved in “elbow-shoulder” interactions between helices A’B’ and C’D’ of the gating ring in CNGA3 and CNGB3 are shown in green. Residues involved in cGMP binding in TAX-4 are shown in orange. Residues involved in CaM binding in CNGB1 are boxed in magenta. Residues that are in close contact with L-*cis*-diltiazem in CNGA1 and CNGB1 are highlighted with a green background. Missense DAMs in CNGA3 and CNGB3 are highlighted with a yellow and brown background, respectively. The C-terminal leucine zipper (CLZ) domains of CNGA1 and CNGA3 are boxed in green. hs: *Homo sapiens*; ce: *C. elegans*.

**Figure 3.**
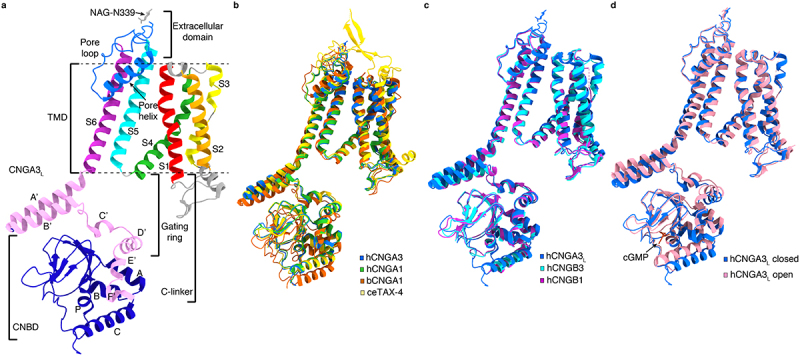
Structures of CNG channel protomers. **a**, protomer structure of apo CNGA3_L_ (PDB accession no. 7RHS) viewed parallel to the membrane. The S1-S6 helices, pore loop and pore helix, C-linker and CNBD are shown in different colors. **b**, superposition of protomer structures of apo human CNGA3 (PDB accession no. 7RHS), human CNGA1 (PDB accession no. 7RH9), bovine CNGA1 (PDB accession no. 7O4H) and *C. elegans* TAX-4 (PDB accession no. 6WEJ). **c**, superposition of protomer structures of apo human CNGA3 (PDB accession no. 7RHS), human CNGB3 (PDB accession no. 7RHS) and human CNGB1 (PDB accession no. 7RH9). **d**, superposition of protomer structures of CNGA3_L_ in apo closed state (PDB accession no. 7RHS) and cGMP-bound open state (PDB accession no. 8EVC).

Prokaryotic (except MloK1) and eukaryotic CNG channel subunits have a high degree of similarity in their primary sequences in the core region (encompassing S1-S6, the C-linker, and the CNBD) but diverge significantly in the N-terminus and beyond the CNBD (see Figure 2 and also Figure 1—figure supplement 6 in ref [27]). It is therefore not surprising that the core regions of different subunits have nearly identical or highly similar structures and the rest of the channels are invisible or poorly visible in the structures (Figure 3a–c). The structure of a CNGA3 protomer is illustrated as an example (Figure 3a). The protomer can be arbitrarily divided into four structural layers from the outside to the inside: an extracellular domain, the transmembrane domain (TMD), the C-linker, and the CNBD (Figure 3a). The TMD includes S1-S6 and the pore-loop and pore helix in between S5 and S6. The C-linker consists of six α-helices, designated as A’–F.’ The C-terminus of helix A’ is connected to the N-terminus of S6 via a 2-amino acid linker. This direct connection is crucial for cyclic nucleotide activation of CNG channels (see later). Helices A’B’ and C’D’ form two antiparallel helix-turn-helix motifs. The A’B’ helices of one subunit associate with the C’D’ helices of their clockwise adjacent subunit through the so-called “elbow-shoulder” intersubunit interactions [16], forming a gating ring that is strategically positioned between the TMD and the CNBD (Figures 1 and 3a). Thus, although there is no domain swapping between the VSLD and PD, there is domain swapping in the C-linker. Since the gating ring is a part of the C-linker, these two structural components are sometimes collectively referred to as C-linker/gating ring from hereon.

The CNBD has the same fold as that of other cyclic nucleotide-binding proteins [48]. It consists of four α helices (designated as A, P, B, and C) and eight β sheets that form a β-roll. Cyclic nucleotides bind in a conserved pocket formed by the helix C and the β-roll and engage in interactions with amino acids highly conserved in all eukaryotic CNG channel subunits (Figure 2). Cyclic nucleotide binding induces conformational changes in the CNBD and many other parts of the channel, including the C-linker/gating ring and S6 (see e.g. Figures 1 and 3d). An obvious change is that the open channel is shorter than the closed channel, with the C-linker/gating and CNBD moving toward the TMD by ~6 Å (Figure 1). Detailed conformational changes are described later.

## Structural basis of ion selectivity, permeation, and block

### Ion conduction pathway

**Figure 4.**
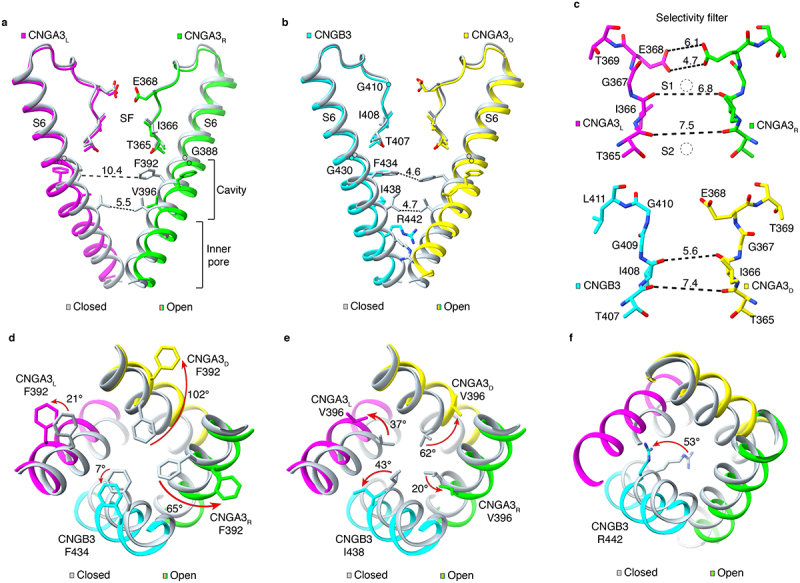
The ion conduction pathway. **a, b**, comparison of the structures of the pore loop and S6 in closed (PDB accession no. 7RHS) and open (PDB accession no. 8EVC) CNGA3/CNGB3 channels. For clarity, only two opposing subunits are shown in each panel. The ion conduction pathway (consisting of the SF, the central cavity and the inner pore) goes through the center. The Cα positions of the hinge glycine are marked by open circles. **c**, close-up views of the SF of the open CNGA3/CNGB3 channel shown in **a** and **b**. In **a-c**, distances (in Å) between the atoms are measured as the center-to-center distance of two diagonally opposed atoms. Open circles represent Ca^2+^-binding site 1 (S1) and site 2 (S2) found in structures of CNGA1/CNGB1 [29]. **d-f**, comparison of S6 and gate residues in closed and open CNGA3/CNGB3 channels, viewed from extracellular side. Arrows indicate motions of the side chains of the specified gate residues, showing the opening of the cavity gate (**d** and **e**) and the arginine gate (**f**). The rearrangements of the cavity gate-forming residues differ from subunit to subunit (**d** and **e**).

Vertebrate CNG channels conduct inward Na^+^ and Ca^2+^ currents under physiological conditions. The ion conduction pathway can be divided into three parts: the SF, the central cavity, and the inner pore. The pore loop forms the SF, and S6 forms the central cavity and inner pore (Figure 4). The ion conduction pathway is symmetrical along the pore axis in homomeric CNG channels but is asymmetrical in native CNGA/CNGB channels owing to the presence of CNGB. Although the amino acid sequence of the pore loop and S6 of CNGB is very similar to that of CNGA, there are critical differences (Figure 2). For example, a conserved glutamate (E368 in CNGA3) in the pore loop of CNGA (aspartate in CNGA4) is replaced by glycine in CNGB, and a conserved glycine in S6 of CNGA (G400 in CNGA3) is substituted by arginine in CNGB. Due to these substitutions and other changes, CNGB subunits greatly affect the native CNG channel ion selectivity, permeation, and block [4].

### Non-selective monovalent cation selectivity

Vertebrate CNG channels are nonselective for group I monovalent cations Li^+^, Na^+^, K^+^, Rb^+^, and Cs^+^ [4,49–59]. The SF is formed by four highly conserved amino acids in the pore loop: TIGE in CNGA subunits (TVGD in human CNGA4) and TIGG in CNGB subunits (Figure 2). In cone CNGA3/CNGB3, they are T365, I366, G367, and E368 of CNGA3 and T407, I408, G409, and G410 of CNGB3. E368 projects its side chain to the SF lumen, which is also lined by the backbone carbonyl oxygens of T365, I377, and G378, the hydroxyl side chain of T376, and their equivalents in CNGB3 (Figure 4c). In fully-open structures of TAX-4, CNGA1, and CNGA3/CNGB3 [24,28,29,37], the side chains of the SF glutamate form the narrowest constriction (4.5–4.7 Å in diameter) of the SF. Computational analyses of the open-state structure of TAX-4 show the highest water density at the glutamate constriction, suggesting that Na^+^ and K^+^ ions can readily permeate this constriction [28]. The rest of the SF is wider (>5.6 Å). The central cavity and the inner pore are even wider (Figure 4a,b).

The physicochemical characteristics of the SF explain why CNG channels are nonselective for monovalent cations. Being at the external entrance of the SF, the electronegative glutamate is well positioned to attract and directly interact with external Na^+^ and K^+^ ions and repel Cl^−^ ions. Neutralizing this glutamate by mutagenesis markedly decreases the inward current and renders the mutant channels outwardly rectifying [60–63]. A ring of four glutamate carboxylates serves as surrogate water molecules to dehydrate and rehydrate permeating cations. The electronegativity and relatively wide dimension of the SF thus allow partially hydrated Na^+^ and K^+^ ions (<5 Å in diameter) to pass with ease, giving rise to the poor monovalent cation selectivity.

In TAX-4 and CNGA1 open-state structures, elongated and diffuse cryo-EM densities are observed at the center of the SF below the glutamate side chains [24,29]. As no other cations are present in the samples, these densities presumably represent Na^+^ ions captured at different positions as they permeate through the SF.

### Ca^2+^ permeation and block

Vertebrate CNG channels, whether homomeric or heteromeric, native or heterologously expressed, are actually more permeable to Ca^2+^ than to Na^+^ [4,64–68]. Thus, vertebrate CNG channels conduct substantial inward Ca^2+^ currents at physiological concentrations [4], which is important for proper phototransduction and olfactory transduction and their regulation by Ca^2+^ [3–6]. Paradoxically, Ca^2+^ (as well as Mg^2+^) blocks monovalent cation currents with a wide ranging apparent affinity of 3–500 μM in different types of CNG channels [4,12,59–61,65,69–72]. Due to the dual action of Ca^2+^, the mean single-channel conductance of rod CNG channels is only ~0.1 pS under physiological conditions [12]. This very low single-channel conductance gives rise to an excellent signal-to-noise ratio and aligns well with the ability of rod photoreceptors to detect a single photon [73].

Functional mutagenesis shows that the SF glutamate is essential for Ca^2+^ permeation and Ca^2+^ block, as neutralizing this glutamate virtually abolishes Ca^2+^ currents and Ca^2+^ block of monovalent cation currents [60,61,70,71]. One might expect that Ca^2+^ is coordinated by the glutamate carboxylates. However, structures of wild-type (WT) human CNGA1 and a mutant carrying a glutamate-to-glutamine (E→Q) mutation obtained in various ionic conditions reveal that Ca^2+^ binds to two distinct and well-resolved sites below the glutamate: a primary site (site 1) formed by the carbonyl oxygen atoms of I363 (I366 in CNGA3) and a secondary site (site 2) formed by the carbonyl and hydroxyl groups of T362 (T365 in CNGA3) [29] (Figure 4c). In the E→Q mutant channel Ca^2+^ binding to site 1 is completely absent and Ca^2+^ binding to site 2 becomes much weaker. These structures explain how the SF glutamate plays three key roles in Ca^2+^ permeation and block [29]: (1) The glutamate carboxylates attract external Ca^2+^ ions and dehydrate them, allowing Ca^2+^ to enter deeper into the SF. (2) The glutamate carboxylates help coordinate Ca^2+^ binding to site 1 through electrostatic interactions. This site has a higher affinity for Ca^2+^ than site 2 does and is likely the blocking site. (3) Electrostatic repulsion between Ca^2+^ ions in the SF, site 1, and site 2 drives Ca^2+^ permeation.

The SF of CNGA1/CNGB1 and CNGA3/CNGB3 channels differs from homomeric CNGA channels in two aspects. (1) The SF glutamate in CNGA is replaced by glycine in CNGB. Thus, heteromeric channels have an asymmetric ring of three instead of four negative charges at the SF external entrance [30,33,34,37]. (2) A positively charged residue in the pore helix (K841 in human CNGB1 and R403 in human CNGB3) projects its side chain toward the SF entrance [30,33,34,37]. Both of these changes would reduce the electronegativity of the SF and weaken Ca^2+^ binding to site 1 in heteromeric CNGA/CNGB channels. This would explain why Ca^2+^ block is much weaker in CNGA1/CNGB1 channels than in CNGA1 channels [4,33,59]. Further supporting the key role of the SF glutamate, Ca^2+^ block of a heteromeric channel formed by CNGA1 and a mutant CNGB1 carrying a glycine-to-glutamate mutation is much enhanced [33].

The structures of CNGA3/CNGB3 further show that R403 in CNGB3 forms hydrogen bonds with the backbone carbonyl oxygens of two glycine residues that line the SF [34,37]. These interactions would probably further weaken Ca^2+^ binding to site 1, which would reduce Ca^2+^ dwell time in the SF and thereby facilitate Ca^2+^ conduction. On the other hand, the analogous amino acid in CNGB1, K841, is not engaged in these interactions [30,33]. This subtle structural difference in the SF of CNGA1/CNGB1 and CNGA3/CNGB3 channels may be a contributing factor to why the fractional current carried by Ca^2+^ is higher in cone CNG channels than in rod CNG channels [4,74]. These hypotheses await functional mutagenesis tests.

### The selectivity filter is not a gate

Before the publication of open- and closed-state structures of TAX-4 in 2020 [28], it was generally accepted that the SF is also the activation gate of CNG channels [2,6,24,75]. This view was based on state-dependent block by Cd^2+^ and state-dependent cysteine modification of CNG channel pores by Ag^+^ or methanethiosulfonate compounds [76–80]. Although cysteine modification results, especially those with Ag^+^, need to be interpreted with care and caveat, many of these studies demonstrated, convincingly, that CNG channels did not have a gate in the inner pore near the cytoplasmic end of S6-like voltage-gated K^+^ channels do [81]. Based on this conclusion and the state of knowledge at the time, it was logical and reasonable to postulate that the activation gate was located in the SF. However, the structures of TAX-4 show that the SF is virtually identical in open and closed states [28]. Subsequent closed, intermediate, partially open, and fully open structures of CNGA1, CNGA1/CNGB1, and CNGA3/CNGB3 all show that the SF does not undergo significant conformational changes during ligand activation [29,33,37]. These structures in aggregate unequivocally demonstrate that the SF is not an activation gate in eukaryotic CNG channels.

### Cavity gate and arginine gate

Where is the CNG channel activation gate if the SF is not it and there is no inner (lower) gate? Structures of closed and open TAX-4 reveal that there is a new gate in the central cavity [28]. This cavity gate is also subsequently found in closed- and open-state structures of CNGA1, CNGA1/CNGB1, and CNGA3/CNGB3 [29,33,34,37]. The cavity gate is formed by two conserved hydrophobic amino acids in S6 (Figure 2); in CNGA3/CNGB3, they are F392 and V396 in CNGA3 and F434 and I438 in CNGB3 (Figure 4a,b,d,e). F392 and F434 are located in the middle of the membrane and immediately below the SF, and V396 and I438 are one α-helical turn down. The side chains of these residues project to the pore lumen in the closed channel and form tight, hydrophobic constrictions (Figure 4a,b,d,e). Computational analyses suggest that this double-barrier hydrophobic seal completely occludes ion conduction [28]. In the open channel, the side chains swing to the side or back of the pore, greatly widening the pore to allow conduction of fully hydrated Na^+^, K^+^, and Ca^2+^ ions (Figure 4a,b,d,e). A hydrophobic cavity gate is also observed in the structures of bacterial LliK, MloK1, and SthK CNG channels [21,25,27,31], indicating that it is a structural feature conserved in prokaryotic and eukaryotic CNG channels.

Structures of CNGA1/CNGB1 and CNGA3/CNGB3 reveal another unexpected new gate in the vertebrate CNG channels [30,33,34,37]. This gate is formed by an arginine in S6 of CNGB (R880 in CNGB1 and R442 in CNGB3) and is located one α-helical turn below the lower cavity gate (Figures 2 and 4b,f). It is referred to as the arginine gate. In the closed channel, the arginine projects its side chain directly into the ion conduction pathway, creating a positively charged barrier for permeating cations (Figure 4b,f). In the open channel, it swings to the side and points tangentially to but not completely away from the pore (Figure 4b,f). Replacing R880 with glycine increases the single-channel conductance of CNGA1/CNGB1 by ~60% [33], indicating a critical role of the arginine gate in modulating ion conduction in heteromeric CNG channels. The arginine gate may contribute to the flickery single-channel gating of native rod and cone CNG channels and heterologously express CNGA/CNGB channels [4,12]. Consistent with this hypothesis, flickery gating behavior is much less pronounced in homomeric CNGA channels, where the arginine is replaced by glycine or serine (Figure 2).

Thus, structural studies have uncovered two gates in heteromeric CNGA/CNGB channels: a cavity gate that constitutes the main activation gate and an auxiliary arginine gate that modulates ion conduction. Why rod and cone CNG channels have two gates remains to be elucidated [9]. One might speculate that having two gates may be related to the fidelity, dynamics, or adaptation of photoreceptors’ response to light. It is noted that R442 in CNGB3 is substituted by glutamine in some animal species that are dichromatic or monochromatic [9,30,34]. The physiological relevance of this R/Q polymorphism remains to be further investigated.

### Block by L-cis-diltiazem

Vertebrate CNG channels can be blocked by membrane-permeable L-*cis*-diltiazem (DTZ) [4,12,55,82,83]. This block takes place from the cytoplasmic side and is voltage-dependent [4,83–85], indicative of a binding site in the pore. The affinity of DTZ for native and heterologously expressed heteromeric CNG channels is much higher than the affinity for homomeric channels [4,59,83,85,86]. Two different structures of the DTZ-bound CNGA1/CNGB1 are obtained in the presence of cGMP [33]. In one structure, the cavity gate is partially open, with DTZ bound at the gate. In another structure, the cavity gate is closed, with the DTZ bound above the gate. In the two structures, DTZ binds to cavity gate residues and other hydrophobic residues through van der Waals interactions, albeit in different orientations and combinations. The presence of CNGB1 creates an asymmetrical but suitable space for DTZ to snuggle in.

## Structural basis of voltage insensitivity

**Figure 5.**
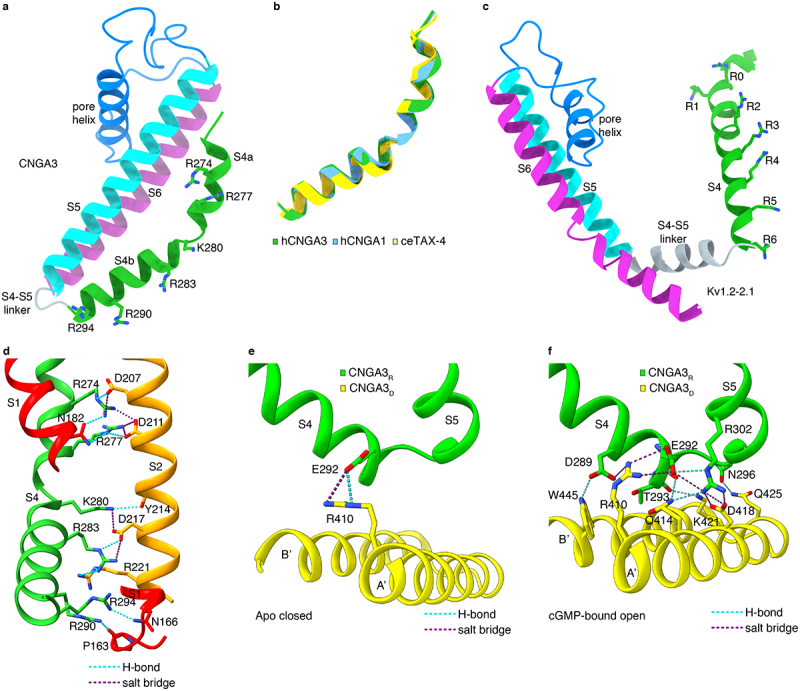
An unusual voltage-insensitive VSLD. **a-c**, comparison of structures of the indicated regions of CNG channels and a Kv1.2–2.1 chimaera, viewed parallel to the membrane. **a**, structure of S4-S6 of CNGA3, showing a segmented S4 and a short and flexible S4-S5 linker (PDB accession no. 7RHS). S4 positive charges are indicated. **b**, superposition of S4 of human CNGA3, human CNGA1 (PDB accession no. 7RH9) and *C. elegans* TAX-4 (PDB accession no. 6WEJ). **c**, structure of S4-S6 of a Kv1.2–2.1 chimaera, showing a continuous α helical S4 and a long and α helical S4-S5 linker (PDB accession no. 2R9R). Positive charges R0–R6 correspond to R287, Q290, R293, R296, R299, K302 and R305, respectively [88]. **d**, interactions of CNGA3 S4 positive charges with partner residues in S1 and S2 (PDB accession no. 7RHS). **e, f**, interactions between S4-S5 and helices A’B’ of the gating ring in closed (PDB accession no. 7RHS) and open (PDB accession no. 8EVC) CNGA3/CNGB3 channels.

S4 serves as the voltage sensor in VGICs and moves in response to membrane potential changes. The CNG channels have an S4 similar to that of VGICs in primary sequence, with multiple regularly spaced positively charged amino acids (Figure 2). However, eukaryotic CNG channels are not activated by membrane potential changes in the absence of cyclic nucleotides, and their ligand-activated currents show little or very weak voltage dependence [4,6,10,24,49,50,52]. Moreover, the CNG channels show little voltage-dependent gating currents [87]. The structures of eukaryotic CNG channels reveal several unique features of their VSLDs that can satisfactorily explain the lack of voltage-dependent gating. Take CNGA3’s VSLD as an example:

First, CNGA3’s S4 consists of two sub-segments, S4a and S4b, with a break in between. S4b is a typical α helix, and S4a is a 3_10_ helix (Figure 5a). S4 in CNGA1 and TAX-4 has a similar structure (Figure 5b). This segmented structure is in sharp contrast to the continuous α helical structure of VGIC S4, as exemplified by K_v_ channel S4 [88,89] (Figure 5c). The short loop between S4a and S4b may act as a “cushion” to reduce voltage-induced conformational changes of S4.

Second, S4b tilts at a shallow angle from the plane of the membrane, thus its three arginine residues are poorly positioned to sense membrane voltage (Figure 5a). In contrast, S4 of Kv channels tilts at a sharp angle [88,89] (Figure 5c) so that its positive charges are well positioned to react to membrane voltage.

Third, the six positive charges on CNGA3 S4 all form either salt bridges or hydrogen bonds with negative charges and other residues in S1-S2-S3 (Figure 5d). These interactions restrict the mobility of S4. Were S4 to move in response to membrane voltages, these interactions would be disrupted and would not be replaceable because of the segmented structure of S4. S4 movement is thus energetically highly unfavorable and indeed prohibitive. Five of the six positive S4 charges and seven of their interacting residues in S1-S2-S3 (including N182, D207, D211, Y214, D217, R221, and T259 in CNGA3) are conserved in CNGA subunits (Figure 2), heightening the robustness and importance of S4/S1-S2-S3 interactions.

Fourth, a typical S4-S5 linker in VGICs is a 10- to 12-amino-acid α-helix running parallel to the plane of the membrane that interacts with S6, an interaction crucial for voltage-dependent gating [88,89] (Figure 5c). The CNGA3 S4-S5 linker is only four amino acids long and does not interact with S6 owing to the non-domain-swapped topology of the VSLD and PD. Instead, it forms a short loop such that S4, S5, and the S4-S5 linker adopt a helix – turn–helix motif (Figure 5a). This motif interacts with the gating ring, further constraining S4. This interaction is relatively weak in the closed channel but very strong in the open channel, effectively immobilizing S4 (Figure 5e, f).

As described in more detail below, the VSLD of CNG channels plays a critical role in ligand gating. Hereditary missense mutations of four conserved CNGA3 S4 arginines (R274, R277, R283, and R290) are linked to achromatopsia [90–95]. The mutant channels do not show cGMP-induced currents [96–98]. Similarly, neutralization of each of these four arginines in bovine CNGA1 S4 and the first two arginines in human CNGA1 S4 also disrupts channel activation [63,87]. Thus, instead of serving as a voltage-sensor, S4 contributes critically to ligand gating of CNG channels.

A critical role of S4 in ligand gating is also revealed in SthK, a bacterial CNG channel SthK activated by both membrane depolarization and cyclic nucleotide binding [31,32,99–101]. In SthK, S4 also interacts with S1-S2-S3 [31]. The structure of WT SthK is closed even when bound with cAMP [27]. Mutations that disrupt S4 interactions with S1-S2-S3 increase channel activity and enable the determination of structures of the mutant channels in intermediate and open states in the presence of cAMP [31]. These structures suggest that S4 inhibits ligand activation of SthK and its displacements allow for more efficient ligand gating.

## Structural mechanisms of ligand activation

### Opening of the cavity gate

**Figure 6.**
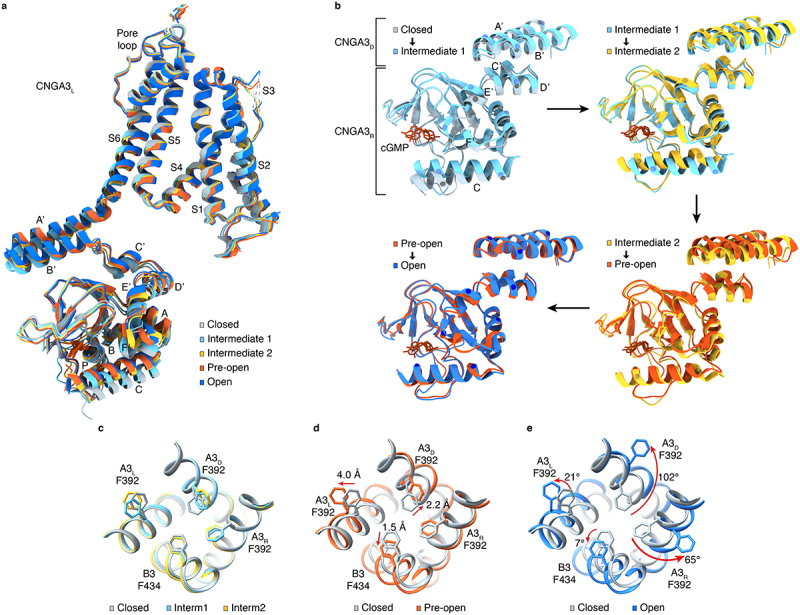
Incremental conformational changes and gradual opening of the cavity gate. **a**, superposition of protomer structures of cGMP-bound CNGA3_L_ in five different states [37]. **b**, Pairwise superposition of the CNBD and C-linker/gating ring of CNGA3_R_ and CNGA3_D_ in closed versus intermediate 1, intermediate 1 versus intermediate 2, intermediate 2 versus pre-open, and pre-open versus open states. The Cα positions of some select residues are marked to facilitate visualization of local conformational changes. **c-e**, comparison of S6 and cavity gate-forming F392/F434 in the five different states shown in (**a**), viewed from extracellular side. Arrows indicate motions of the side chains of the specified gate residues, showing gradual, subunit-specific and state-dependent rearrangements. The figures panels are adopted from Hu et al. [37].

**Figure 7.**
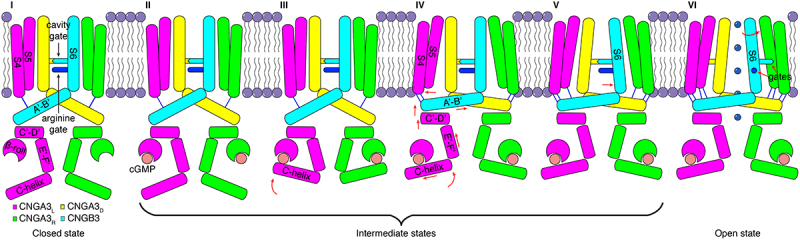
Model of allosteric gating of CNGA3/CNGB3 by cGMP. The schematic is based on the closed, intermediate, pre-open and open state structures obtained in POPG/POPC nanodiscs and depicts stepwise conformational rearrangements in several key regions during channel opening. The conformational changes triggered by cGMP binding to the β-roll and C-helix of the CNBD propagate to the pore-forming S6 through helices E’F’ of the C-linker and helices A’B’C’D’ of the gating ring, ultimately resulting in the opening of the cavity gate and arginine gate. The sequential movement of different regions is highlighted in red. Blue lines indicate interactions between S4-S5 and helices A’B.’ This figure is adopted from Hu et al. [37].

How do cyclic nucleotides activate CNG channels? The cavity gate is tightly closed in the absence of activating ligands and is >50 Å away from the cyclic nucleotide-binding site. To allosterically open this gate, the conformational changes triggered by cyclic nucleotide binding must propagate from the CNBD to the TMD. Structures of eukaryotic CNG channels in closed, intermediate, and open states [28,29,33,37] provide a comprehensive view of such conformational changes. In particular, the structures of human CNGA3/CNGB3 in closed, transition, pre-open and open states (Figure 6) provide especially lucid details on the conformational trajectory of activation of a vertebrate CNG channel by its natural ligand [37]. In broad terms, cGMP binds to the CNBD and causes it to move toward the membrane; this causes the C-linker/gating ring to move closer to the TMD and S4 and S5 to move away radially from the pore; these movements lead to dilation and rotation of S6 and opening of the cavity gate. More specifically, the spatial allosteric conformational wave can be arbitrarily simplified into the following steps (Figures 6 and 7):

1. In the closed state, the CNBD is in a relaxed resting conformation with its C-helix in a “down” position. cGMP binding causes the C-terminal end of the C-helix to move upward. Helices A, B, and P and the β-roll of the CNBD also move toward cGMP. In this intermediate state, the CNBD is more compact, like a closed clamshell.
2. The C-helix moves up further (particularly its N-terminal end), rotates along its own axis, and slides leftward. It is now in an “up” position. These movements cause the E′F′ helices of the C-linker to move vertically toward the TMD by >5 Å.
3. The large upward movement of helices E′F′ causes helices C′D′ of the C-linker/gating ring to move closer to the TMD by ~3 Å. Because of the “elbow-shoulder” interactions between helices A’B’ and C’D’, helices A’ and B’ both undergo dramatic and complex conformation rearrangements. They tilt, rotate, slide, and move toward the TMD by as much as 5 Å. As a result, the N-terminal end of helix A′ that connects to S6 expands radially by 6–7 Å in a pre-open state.
4. The movements of the C-linker/gating ring are transmitted to the TMD through two coupling mechanisms. First, helix A’ of the gating ring is connected to S6 via a two-amino acid linker. Thus, helix A’ movements are transmitted to S6 through direct pulling. Second, helices A’B’ of the gating ring interact with the S4-linker-S5 motif of an adjacent subunit. These interactions vary between different subunit pairs and in different states, relatively weak in the closed state and much stronger in the open state [37] (Figure 5e,f). Because of these interactions, helix A’ movements cause S4 and S5 to move away from the pore, which then causes S6 to dilate.

The structural revelation of the essential role of the C-linker/gating ring in the allosteric coupling between the CNBD and S6 is in good agreement with extensive functional results demonstrating C-linker/gating ring’s critical role in ligand gating [4,6,10,11,102–107].

(5) As a result of direct pulling by helix A’ of the gating ring and outward expansion of S4 and S5, S6 undergoes incremental dilation and rotation, resulting in a gradual opening of the cavity gate. A hinge point for S6 dilation and rotation is G388 in CNGA3 and G430 in CNGB3, which are located one α-helical turn above the cavity gate-forming F392/F434 (Figure 3a,b). This glycine is conserved in most CNG channel subunits but, curiously, the analogous residue is alanine in CNGA4 (Figure 2).

The actual conformational rearrangements of CNGA3/CNGB3 are much more complex and are subunit-specific, localized, incremental, and sequential. They can be more clearly visualized and conceptualized in a movie showing a continuum of 13 different three-dimensional (3D) maps that correspond to channels in closed, intermediate, pre-open, and open states (Supplementary Movie 1).

Asymmetrical subunit-specific conformational changes during gating are also observed in CNGA1/CNGB1 [33] where two apparently partially open states are observed. In these structures, only CNGA1_L_ and CNGA1_D_ open their gate residues. Two partially open structures are also obtained for SthK R120A [31]. These structures show an activated CNBD and C-linker/gating ring and an open but narrower pore. A pre-open state is observed in SthK Y26F, which has a nearly fully activated CNBD and C-linker/gating ring but a closed pore [31]. These structures are in general agreement with the gating steps and model described above. Why these structured are captured in these states is yet to be examined, but different lipid compositions may be a contributing factor.

### Opening of the arginine gate

As with the gradual opening of the cavity gate in CNGA3/CNGB3, the arginine gate formed by R442 of CNGB3 also undergoes gradual conformational changes during closed-to-open transitions and opens fully only in the open state [37]. R442 projects directly to the ion conduction pathway in the closed state (Figure 4f) Its side chain adopts a rotamer conformation of up and down orientations. The up orientation diminishes in intermediate states. In the open state, the R442 side-chain rotates from the center position to a side position (Figure 4f). The R442 side chain is stabilized in different orientations and states by interactions with different residues in S6. Rotamer conformations and interactions with S6 are also observed for the arginine gate in the rod CNGA1/CNGB1 channels [9,30,33,34]. The functional importance of a relatively flexible arginine gate in cone and rod photoreceptor CNG channels remains to be elucidated. One possibility is that it contributes to the flicker gating behavior of native CNG channels, as mentioned above.

## Structural basis of modulation

### Modulation by calmodulin

Native and heterologously expressed CNG channels undergo Ca^2+^-dependent desensitization by direct binding of calmodulin (CaM) [4,6]. This modulation is important for light adaptation in photoreceptors [4,6,108–110]. Recent studies have begun to examine the structural basis of the CaM modulation of CNGA1/CNGB1 [36,111]. CNGB1 has two CaM binding sites: an IQ-like motif in the N-terminus (CaM1) and another site in the C-terminus (CaM2) (Figure 2) [112]. NMR structures show that peptides containing either the CaM1 or CaM2 site of bovine CNGB1 bind the N-lobe of Ca^2+^/CaM through predominantly hydrophobic interactions [111]. The cryo-EM structure of bovine CNGA1/CNGB1 in complex with Ca^2+^/CaM shows that the C-lobe of Ca^2+^/CaM is bound to the CaM2 site, but the N-lobe is invisible [36]. Comparison of the structures of apo CNGA1/CNGB1 with and without CaM shows that while Ca^2+^/CaM binding does not significantly alter the ion conduction pathway, it produces some global conformational changes, including a tilting of the CLZ domains of CNGA1, an overall contraction of the cytosolic and transmembrane regions, and a slight widening of SF [36]. Since the CaM2 site does not affect CaM regulation of rod CNG channels, it is postulated that the CaM binding at this site serves a structural purpose by stabilizing the channel in the closed state [36].

### Modulation by lipids

The CNG channels are modulated by membrane lipids [4,6]. For example, native and heterologously expressed CNG channels are inhibited by diacylglycerol, phosphatidylinositol (4,5)-trisphosphate and phosphatidylinositol (3,4,5)-trisphosphate [113–119]. On the other hand, the bacterial CNG channel SthK is potentiated by anionic lipids [32,100]. Lipids are observed in SthK structures, including anionic lipids [27,32]. The structure of SthK bound with 1-palmitoyl-2-oleoyl-phosphatidic acid (POPA) shows that POPA binds to a groove in the membrane inner leaflet formed by two adjacent S6 helices and its negatively charged headgroup electrostatically disrupts an intersubunit salt bridge between S5 and S6 that stabilizes the closed-state [32]. This modulation mechanism is likely absent in the eukaryotic CNG channels since the described S5-S6 salt bridge is absent and no lipid is observed in the corresponding groove. However, the residues that form the S5-S6 salt bridge responsible for lipid modulation in SthK are conserved in sequence and structure in eukaryotic HCN channels [120–122], which are potentiated by negatively charged lipids. Breaking the S5-S6 salt bridge in HCN2 greatly weakens its potentiation by phosphatidic acid [32].

Abundant non-protein densities are observed in cryo-EM density maps of the eukaryotic CNG channels [28–30,33,37]. Some of these densities likely represent lipids. Indeed, a variety of lipids are detected in a protein sample of human CNGA3/CNGB3 reconstituted in the detergent glycol-diosgenin (GDN) [37]. Strikingly, structures of CNGA3/CNGB3 in GDN or lipid nanodiscs containing 1-palmitoyl-2- oleoyl-sn-glycero-3-phosphoglycerol (POPG) and 1-palmitoyl-2-oleoyl-sn-glycero-3-phosphocholine (POPC) show that all the channels in GDN and most (>92%) channels in POPG/POPC nanodiscs are in closed or intermediate states, even though the channels are fully bound with cGMP, and that the open state exists only in POPG/POPC nanodiscs, with <5% of the channels [37]. These results suggest that lipids play a critical role in shaping the energetic landscape of ligand activation of vertebrate CNG channels.

## Structural basis of channelopathy

Numerous inherited mutations, including frameshift, deletion, insertion, and missense mutations, in rod and cone CNG channels are linked to degenerative visual disorders [4,6,7,91,123]. Most mutations cause loss-of-function (LOF) but some mutations produce gain-of-function (GOF). The structures of eukaryotic CNG channels allow the mapping of the 3D positions of most of the exonic disease-associated mutations (DAMs), allowing for a better understanding of how DAMs alter channel structure and function. Obtaining structures of mutant channels carrying DAMs, on the other hand, may shed further light on their pathogenic mechanisms and provide different perspectives into the molecular mechanisms of CNG channel structure and function.

An example of this line of research is the recent determination of structures of a mutant TAX-4 carrying a missense DAM found in CNGA3 [35]. The mutation is R410W in CNGA3 and R421W in TAX-4. The R410W mutation produces complete color blindness [90,91] and is reportedly a LOF mutation as cells expressing the mutant channel have no cyclic nucleotide-induced currents [98]. The mutated arginine is conserved in CNGA1, CNGA2, CNGA3, and TAX-4, but intriguingly, the analogous position is aspartate in CNGA4 (Figure 2). R410 is located in the gating ring. Structures of CNGA3/CNGB3 reveal that in the closed-state, R410 interacts with a negative charge in S4 (E292) [34,37] (Figure 5e). This interaction is also present in TAX-4, where R421 and E298 forms a salt bridge [35]. The structure of TAX-4 R421W in the presence of cGMP is identical to that of cGMP-bound WT TAX-4, indicating that the R421W mutation does not affect cGMP activation of TAX-4 [35]. Surprisingly, the structures of TAX-4 R421W in the absence of cGMP reveal not only a normal closed state but also a normal open state. These structures indicate that the R421W mutation disrupts the R421-E298 salt bridge, destabilizes the closed state, and renders the mutant channel spontaneously open. Functional experiments confirm that the R421W/R410W mutation increases the spontaneous activity of TAX-4 and human CNGA3/CNGB3 channels. Moreover, expression of either TAX-4 R421W or CNGA3/CNGB3 R410W mutant channels in cell lines causes cell death. These results together suggest a new possibility for an apparent LOF phenotype of a mutant channel: the channel is spontaneously active and is thus toxic to cells, and therefore cells with high levels of surface channels die during cell culture; as a result, patch-clamp recordings are biased toward cells containing just a co-transfected fluorescent marker or cells with mainly intracellular channels, and these cells produce little or no currents. This study calls for a reevaluation of some other missense DAMs that have been previously characterized as LOF mutations.

## Perspectives

Structural studies of CNG channels have made great strides in the past 6 years and have provided deep mechanistic insights into CNG channel properties and mechanisms. However, many outstanding questions remain. Some interesting and important questions have been raised in a recent review on structures of rod and cone CNG channels [9]. We highlight some other questions below:

1. What is the structure of the olfactory CNG channel? The native CNG channel in olfactory sensory neurons are formed by CNGA2, CNGA4, and CNGB1b [124], with a stoichiometry of two CNGA2, one CNGA4, and one CNGB1b [125]. Comparative functional studies show that the heterologously expressed channel exhibits native-like biophysical properties only when all three subunits are co-expressed [124,126–128]. Structures of the olfactory CNG channel would provide a framework to address many other questions, including subunit arrangement, similarities and differences with rod and cone CNG channels, and the role of each subunit in shaping the properties of the channel.
2. What is the structural basis of cooperativity? Cyclic nucleotide activation of vertebrate CNG channels is highly cooperative, with a Hill coefficient of 2 to 4, suggesting that two or more cyclic nucleotide molecules are required for full activation [4,6,129–137]. Functional studies on vertebrate CNG channels activated by 1, 2, 3, or 4 CNs [129,130,132,134] show that binding of one CN only slightly increases channel activity and full activation requires binding of four CNs. In the homomeric CNGA2 channel, binding of the second activating ligand is most critical for channel opening, while binding of the third and fourth ligands only drives the channel away from the critical second binding step and thereby stabilizes the open state [134]. On the other hand, binding of the second and the third ligands is highly cooperative in heteromeric CNGA2/CNGA4/CNGB1b channel, where subunit cooperativity is confined to the CNBD, whereas the subunit promotion energies for channel opening are independent [137]. These results raise many questions on how cooperativity is achieved in different CNG channels. A full understanding of the molecular mechanisms of cooperativity of CNG channel gating would benefit greatly from the determination of structures of CNG channels with different numbers of bound ligands.
3. How do native membrane lipids modulate CNG channels? As mentioned above, structures of CNGA3/CNGB3 in GDN and POPC/POPG lipid nanodiscs show that all the channels in GDN are closed and the vast majority of channels in POPC/POPG nanodiscs are either closed or in intermediate states [37]. This is striking since all the channels are fully bound with cGMP. These findings, combined with the seemingly contradicting fact that native and heterologously expressed CNGA3/CNGB3 are robustly activated by cGMP, indicate that native lipids are crucially involved in cGMP activation of CNGA3/CNGB3. Is this also the case for CNGA1/CNGB1 and CNGA2/CNGA4/CNGB1b channels? Which lipids are critical for cyclic nucleotide activation of CNG channels? Where do these lipids bind and how do they stimulate or inhibit CNG channel activity? Structures of CNG channels reconstituted in different lipid compositions, combined with structure-guided functional mutagenesis, would help answer these questions.
4. How do missense DAMs alter CNG channel structure and function? More than 250 mutations in CNGA3 and more than 160 mutations in CNGB3 are found to cause achromatopsia, and a large number of mutations in CNGA1 and CNGB1 are found to cause retinitis pigmentosa [8]. Most of the DAMs are deletion, frameshift, or insertion mutations that produce truncated channel subunits that lack key functional domains. The reasons underlying their pathogenicity are usually obvious at the channel level, so these mutations are uninformative with respect to understanding CNG channel properties and mechanisms. However, many DAMs are missense mutations. The unexpected finding that a presumed LOF missense DAM renders the mutant channel spontaneously open [35] raises the question of whether some other DAMs also have the same effect. It would be especially interesting to examine how missense DAMs of amino acids located in regions critical for coupling ligand binding to pore opening affect channel structure and gating.

Addressing these questions would require multipronged investigations that combine structural approaches with functional, biochemical, and bioinformatical, single molecule, imaging, and computational approaches. The rapid progress of structural studies of CNG channels is a harbinger of more exciting research ahead.

## Supplementary Material

Supplemental Material

## Data Availability

Data sharing is not applicable to this article as no new data were created or analyzed in this study.
