# Supplementary material for: Structural basis of properties, mechanisms, and channelopathy of cyclic nucleotide-gated channels
Source: Channels (Austin). 2023 Oct 31;17(1):2273165. doi: 10.1080/19336950.2023.2273165 (PMC10761061; doi:10.1080/19336950.2023.2273165)
Supplement: Supplemental Material [file KCHL_A_2273165_SM2554.zip › Supplementary files/Supplemental_Online_Material.docx]

**Supplemental Online Material**

**Supplementary Movie 1. Conformational changes of cGMP-bound CNGA3/CNGB3 in POPG/POPC nanodiscs.** The movie is generated from 3D variability analysis and shows a continuum of 13 different 3D density maps generated from 99.4% of the particles used to produce the five states in POPG/POPC nanodiscs. Each subunit is color coded as in Figure 1. The channel is first viewed parallel to the membrane (side view) and then from the intracellular side (bottom-up view). Notice that each subunit undergoes different movements. This movie is adopted from Hu et al. [39]
